# The genome sequence of a coronate scyphozoan jellyfish, *Nausithoe racemosa* (Komai, 1936) (Coronatae: Nausithoidae), and a metagenome-assembled genome of the associated cyanobacterium *Moorena producens*

**DOI:** 10.12688/wellcomeopenres.27239.1

**Published:** 2026-07-28

**Authors:** Michael N. Dawson, Graeme Oatley, Elizabeth Sinclair, Eerik Aunin, Noah Gettle, Camilla Santos, Michael Paulini, Haoyu Niu, Victoria McKenna, Rebecca O’Brien, Wellcome Sanger Institute Tree of Life Management, Samples and Laboratory team, Wellcome Sanger Institute Scientific Operations: Sequencing Operations, Wellcome Sanger Institute Tree of Life Core Informatics team, EBI Aquatic Symbiosis Genomics Data Portal Team, Aquatic Symbiosis Genomics Project Leadership

**Affiliations:** 1 Life & Environmental Sciences, University of California, Merced, Merced, California, USA; 2 Tree of Life Programme, Wellcome Sanger Institute, Hinxton, England, UK

**Keywords:** Nausithoe racemosa, coronate scyphozoan jellyfish, genome sequence, chromosomal, Coronatae, microbial metagenome assembly

## Abstract

We present a genome assembly from a specimen of *Nausithoe racemosa* (coronate scyphozoan jellyfish; Cnidaria; Scyphozoa; Coronatae; Nausithoidae). The assembly contains two haplotypes with total lengths of 4 784.66 megabases and 4 868.20 megabases. Most of haplotype 1 (97.34%) is scaffolded into 20 chromosomal pseudomolecules. Haplotype 2 was assembled to scaffold level. The mitochondrial genome has also been assembled, with a length of 13.97 kilobases. From the metagenome data, we recovered one high-quality metagenome-assembled genome.

## Species taxonomy

Eukaryota; Opisthokonta; Metazoa; Eumetazoa; Cnidaria; Scyphozoa; Coronatae; Nausithoidae; *Nausithoe*; *Nausithoe racemosa* (Komai, 1936) (NCBI:txid2976324).

## Background

The coronate medusae (Cnidaria: Scyphozoa: Coronatae) comprise one of two major clades of “true jellyfish” within Scyphozoa, the other being Discomedusae ( Bayha *et al.*, 2010). Coronates are best known as deep-water, mesopelagic jellyfish ( Arai, 1997: p. 5; Mianzan & Cornelius, 1999; OBIS, 2026). Shallow-water species are exceptions, notably in *Nausithoe* and *Linuche* ( Bayha *et al.*, 2010; Molinari *et al.*, 2023). These genera are also unusual among coronates because both include photosymbiotic species ( Djeghri *et al.*, 2019; Hingston *et al.*, 2007).

The phylogeny of coronates is poorly resolved, leaving several questions unanswered ( Bayha *et al.*, 2010). For example: 1. Is Nausithoidae basally branching in the clade? 2. Is *Nausithoe* polyphyletic, with one lineage inhabiting deep-water oceanic habitats and the other inhabiting shallow-water coastal habitats? 3. Are the shallow-water coronates, *Nausithoe* and *Stephanoscyphus* monophyletic? 4. Did the photosymbioses in *Nausithoe* and *Stephanoscyphus* originate independently?

The origin of photosymbiosis in *Nausithoe*, whether or not it is independent of that in *Stephanoscyphus*, is noteworthy because it is clearly independent of another origin of photosymbiosis within the Scyphozoa: in the Rhizostomeae clade Kolpophorae ( Djeghri *et al.*, 2019). In both *Nausithoe* and Kolpophorae, the photosymbiont is a member of the Symbiodiniaceae (Dinoflagellata), often referred to as zooxanthellae. In *Nausithoe*, preliminary sequencing analyses indicate that the zooxanthellar endosymbionts belong to the genus *Cladocopium* (previously *Symbiodinium*) ( LaJeunesse *et al.*, 2018; Higgins-Poling, 2024: p. 32). The holobiont is too poorly studied to say whether *Nausithoe* may form photosymbioses with additional species of Symbiodiniaceae, or whether a single host can harbour more than one dinoflagellate species at a time. These patterns are known among scyphozoans ( Trench & Luong-van Thinh, 1995; Arai, 1997: p. 105).

**Figure 1.**
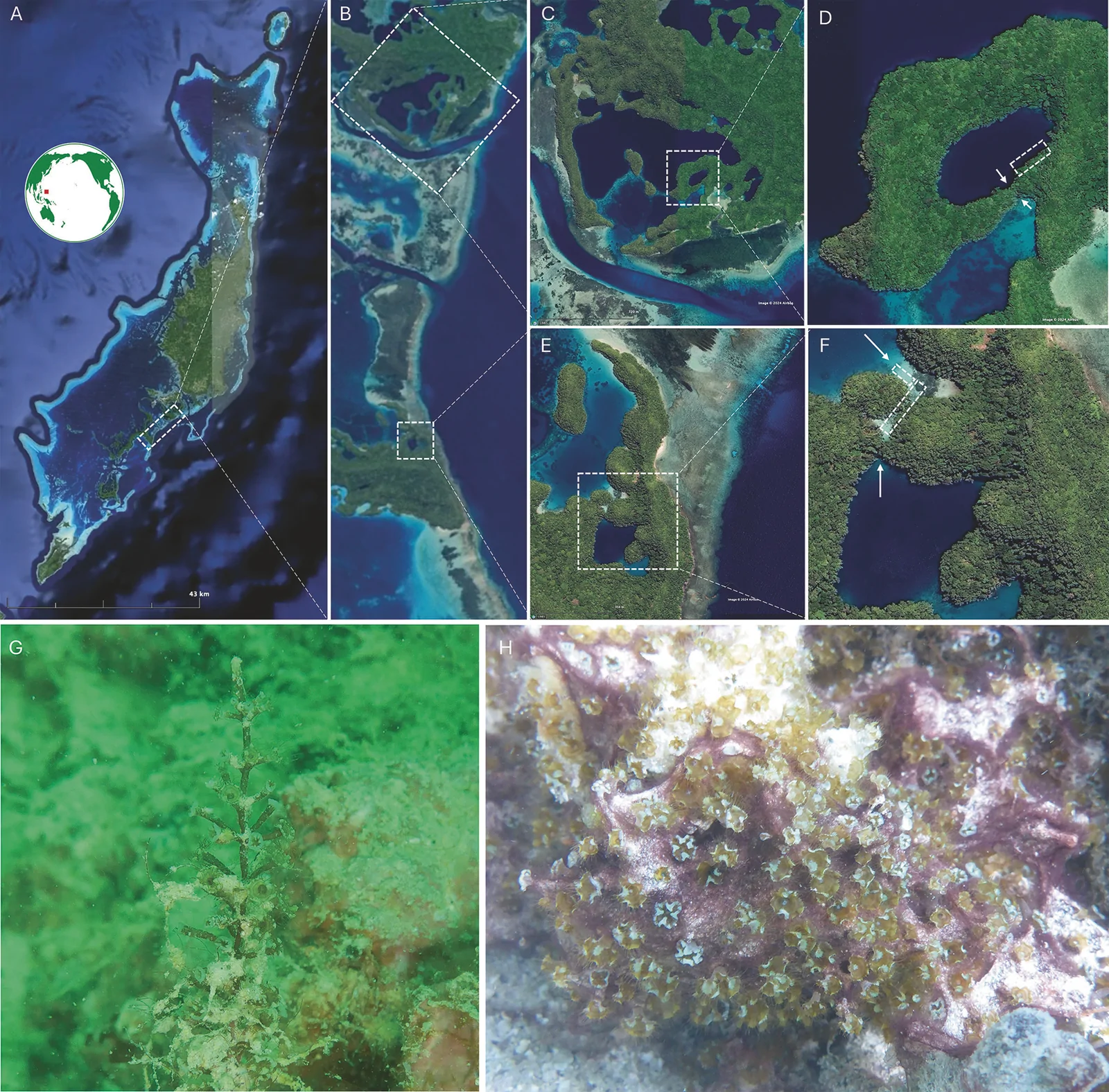
A. Palau, with its tropical western Pacific location indicated by the grey square on the inset world map. **B.** The coral reef and channels forming the eastern entrance to Malakal Harbor, framed by the islands of Auluptagel (above) and Urukthapel (below), each of which contains a locality where *Nausithoe racemosa* polyps have been recorded. **C.** Risong Cove, and **D.** detail of Risong Cove; the dashed box shows where *N. racemosa* polyps have regularly been found over the past two decades and where the specimen was sampled for genome sequencing. **E.** Tab Kukau Cove, and **F.** detail of Tab Kukau Cove; the dashed box shows where *N. racemosa* polyps have regularly been found over the past two decades. In **D** and **F**, the opposing arrows indicate shallow channels through which seawater enters and leaves the coves, and which provide small-boat access during mid-to-high tides. **G.** *N. racemosa* polyps at approximately 10 m depth on a rock wall in Risong Cove. **H.** *N. racemosa* polyps along the edge of a shallow channel leading to Tab Kukau Cove. World map from Pixabay. Satellite imagery from Google Earth. Photographs by M. N. Dawson.

*Nausithoe* is also relevant to questions about life-history evolution in the clade. If Nausithoidae is relatively basal in the clade, close to Linuchidae, this would support the inference that a polyp stage is plesiomorphic for Coronatae, consistent with ancestral state reconstruction for most medusozoans, and that polyps ancestrally reproduce asexually through polydisc strobilation ( Collins, 2002). In that case, the benthic polyp stage was lost from derived deep-water coronates such as *Periphylla* ( Helm, 2018; Jarms *et al.*, 1999). Nausithoid polyps are dendritic in form, often bushy ( Figure 1), and produce many small medusae (10–15 mm bell diameter) at a time ( Kölliker, 1853).

*Nausithoe racemosa* is not known to be of conservation concern. Although not well sampled geographically, initial analyses indicate that it is relatively widespread, with species-level differences arising at scales of 2500 km or more ( Abboud *et al.*, 2018). Colonies contain many polyps, each capable of polydisc strobilation. Its bipartite life history is, as for other metagenic medusozoans, inferred to confer an ability to persist through periods that are unfavourable for medusae ( Arai, 1997: p. 139).

Here, we report a new genome assembly for *Nausithoe racemosa*, the first described genome assembly for a photosymbiotic shallow-water nausithoid, the first for Nausithoidae, and the first for a coronate scyphozoan jellyfish.

## Methods

### Sample acquisition

Specimens of *Nausithoe racemosa* were picked with long forceps and placed in large plastic bags while diving using SCUBA in Risong Cove, Koror State, Palau (latitude 7.1825, longitude 134.2838) on 2022-07-28. They were transported live to the laboratory (Coral Reef Research Foundation, Koror, Palau) within 1 to 2 hours. At the lab, tissues were biopsied and immediately frozen in liquid nitrogen. Samples were subsequently stored and shipped in an ultra-cold dry shipper, and transferred to a − 80 °C freezer at the University of California, Merced, where they were stored before forwarding to the Wellcome Sanger Institute on dry ice. The specimen used for genome sequencing had specimen ID UCALI0000003 and ToLID jsNauRace1. The jsNauRace1 specimen was also used for RNA sequencing.

### Nucleic acid extraction

Protocols for high molecular weight (HMW) DNA extraction developed at the Wellcome Sanger Institute (WSI) Tree of Life Core Laboratory are available on protocols.io ( Howard *et al.*, 2025). The jsNauRace1 sample was weighed and triaged to determine the appropriate extraction protocol. Tissue was homogenised by powermashing using a PowerMasher II tissue disruptor. HMW DNA was extracted using the Manual MagAttract v3 protocol. DNA was sheared into an average fragment size of 12–20 kb following the Megaruptor®3 for LI PacBio protocol. Sheared DNA was purified by automated SPRI (solid-phase reversible immobilisation). The concentration of the sheared and purified DNA was assessed using a Nanodrop spectrophotometer and Qubit Fluorometer using the Qubit dsDNA High Sensitivity Assay kit. Fragment size distribution was evaluated by running the sample on the FemtoPulse system.

RNA was extracted from the jsNauRace1 specimen in the Tree of Life Laboratory at the WSI using the RNA Extraction: Automated MagMax™ *mir*Vana protocol. The RNA concentration was assessed using a Nanodrop spectrophotometer and a Qubit Fluorometer using the Qubit RNA Broad-Range Assay kit. Analysis of the integrity of the RNA was done using the Agilent RNA 6000 Pico Kit and Eukaryotic Total RNA assay.

### PacBio HiFi library preparation and sequencing

Library preparation and sequencing were performed at the WSI Scientific Operations core. Libraries were prepared using the SMRTbell Prep Kit 3.0 (Pacific Biosciences, California, USA), following the manufacturer’s instructions. The kit includes reagents for end repair/A-tailing, adapter ligation, post-ligation SMRTbell bead clean-up, and nuclease treatment. Size selection and clean-up were performed using diluted AMPure PB beads (Pacific Biosciences). DNA concentration was quantified using a Qubit Fluorometer v4.0 (ThermoFisher Scientific) and the Qubit 1X dsDNA HS assay kit. Final library fragment size was assessed with the Agilent Femto Pulse Automated Pulsed Field CE Instrument (Agilent Technologies) using the gDNA 55 kb BAC analysis kit.

The sample was sequenced on a Revio instrument (Pacific Biosciences, California, USA). Prepared libraries with a molarity greater than 2 nM were normalised to 2 nM, and 15 μL was used for making complexes. For libraries with a molarity below 2 nM, the available 10 μL library volume was used without normalisation. Primers were annealed and polymerases were bound to generate circularised complexes, following the manufacturer’s instructions. Complexes were purified using 1.2× SMRTbell beads, diluted to the Revio loading concentration of 200–300 pM, and spiked with a Revio sequencing internal control. The sample was sequenced on a Revio 25 M SMRT cell. SMRT Link software (Pacific Biosciences), a web-based workflow manager, was used to configure and monitor the run and to carry out primary and secondary data analysis.

### Hi-C

***Sample preparation and crosslinking***

The Hi-C sample was prepared from 20–50 mg of frozen tissue of the jsNauRace1 sample using the Arima-HiC v2 kit (Arima Genomics). Following the manufacturer’s instructions, tissue was fixed and DNA crosslinked using TC buffer to a final formaldehyde concentration of 2%. The tissue was homogenised using the Diagnocine Power Masher-II. Crosslinked DNA was digested with a restriction enzyme master mix, biotinylated, and ligated. Clean-up was performed with SPRISelect beads before library preparation. DNA concentration was measured with the Qubit Fluorometer (Thermo Fisher Scientific) and Qubit HS Assay Kit. The biotinylation percentage was estimated using the Arima-HiC v2 QC beads.

***Hi-C library preparation and sequencing***

Biotinylated DNA constructs were fragmented using a Covaris E220 sonicator and size selected to 400–600 bp using SPRISelect beads. DNA was enriched with Arima-HiC v2 kit Enrichment beads. End repair, A-tailing, and adapter ligation were carried out with the NEBNext Ultra II DNA Library Prep Kit (New England Biolabs), following a modified protocol where library preparation occurs while DNA remains bound to the Enrichment beads. Library amplification was performed using KAPA HiFi HotStart mix and a custom Unique Dual Index (UDI) barcode set (Integrated DNA Technologies). Depending on sample concentration and biotinylation percentage determined at the crosslinking stage, libraries were amplified with 10–16 PCR cycles. Post-PCR clean-up was performed with SPRISelect beads. Libraries were quantified using the AccuClear Ultra High Sensitivity dsDNA Standards Assay Kit (Biotium) and a FLUOstar Omega plate reader (BMG Labtech).

Prior to sequencing, libraries were normalised to 10 ng/μL. Normalised libraries were quantified again to create equimolar and/or weighted 2.8 nM pools. Pool concentrations were checked using the Agilent 4200 TapeStation (Agilent) with High Sensitivity D500 reagents before sequencing. Sequencing was performed using paired-end 150 bp reads on the Illumina NovaSeq X.

### RNA library preparation and sequencing

Libraries were prepared using the NEBNext® Ultra II Directional RNA Library Prep Kit for Illumina (New England Biolabs), following the manufacturer’s instructions. Poly(A) mRNA was isolated from 100 ng of total RNA using oligo (dT) beads, converted to cDNA, and uniquely indexed; 14 PCR cycles were performed. Libraries were prepared with an intended fragment size of 100–300 bp. Libraries were quantified, normalised, and pooled equimolarly to a final concentration of 2.8 nM before dilution to 150 pM for loading. Sequencing was carried out on the Illumina NovaSeq X, generating paired-end reads.

### Genome assembly

Prior to assembly of the PacBio HiFi reads, a database of *k*-mer counts ( *k* = 31) was generated from the filtered reads using FastK. GenomeScope2 ( Ranallo-Benavidez *et al.*, 2020) was used to analyse the *k*-mer frequency distributions, providing estimates of genome size, heterozygosity, and repeat content.

The HiFi reads were assembled using Hifiasm in Hi-C phasing mode ( Cheng *et al.*, 2021, 2022), producing two haplotypes. Hi-C reads ( Rao *et al.*, 2014) were mapped to the primary contigs using bwa-mem2 ( Vasimuddin *et al.*, 2019). Contigs were further scaffolded with Hi-C data in YaHS ( Zhou *et al.*, 2023), using the --break option for handling potential misassemblies. The scaffolded assemblies were evaluated using Gfastats ( Formenti *et al.*, 2022), BUSCO ( Manni *et al.*, 2021) and MERQURY.FK ( Rhie *et al.*, 2020).

The mitochondrial genome was assembled using MitoHiFi ( Uliano-Silva *et al.*, 2023).

### Assembly curation

The assembly was decontaminated using the Assembly Screen for Cobionts and Contaminants ( ASCC) pipeline. TreeVal was used to generate the flat files and maps for use in curation. Manual curation was conducted primarily in PretextView and HiGlass ( Kerpedjiev *et al.*, 2018). Scaffolds were visually inspected and corrected as described by Howe *et al*. (2021). Manual corrections included 175 breaks, 211 joins, and removal of 51 haplotypic duplications. This reduced the scaffold count by 3.2%, reduced the scaffold N50 by 4.5%, and reduced the total assembly length by 0.8%. The curation process is described at sanger-tol/curation-resources . PretextSnapshot was used to generate a Hi-C contact map of the final assembly.

### Assembly quality assessment

The Merqury.FK tool ( Rhie *et al.*, 2020) was run in a Singularity container ( Kurtzer *et al.*, 2017) to evaluate *k*-mer completeness and assembly quality for both haplotypes using the *k*-mer database ( *k* = 31) computed prior to genome assembly. The analysis outputs included assembly QV scores and completeness statistics.

The genome was analysed using the BlobToolKit pipeline, a Nextflow implementation of the earlier Snakemake version ( Challis *et al.*, 2020). PacBio reads were aligned to the assembly using minimap2 ( Li, 2018) and processed with SAMtools ( Danecek *et al.*, 2021) to generate coverage tracks. The pipeline runs BUSCO ( Manni *et al.*, 2021) using lineages identified from the NCBI Taxonomy ( Schoch *et al.*, 2020). For the three basal BUSCO lineages, eukaryota, bacteria and archaea, BUSCO genes are aligned to the UniProt Reference Proteomes database ( Bateman *et al.*, 2023) using DIAMOND BLASTp ( Buchfink *et al.*, 2021). The genome assembly is divided into chunks based on the density of BUSCO genes from the closest taxonomic lineage, and each chunk is aligned to the UniProt Reference Proteomes database using DIAMOND BLASTx. Sequences without DIAMOND BLASTx hits are extracted using seqtk and aligned to the NT database using BLASTn ( Altschul *et al.*, 1990). The outputs are consolidated into a BlobDir for visualisation in BlobToolKit. The BlobToolKit pipeline was developed using nf-core tooling ( Ewels *et al.*, 2020) and MultiQC ( Ewels *et al.*, 2016), with containerisation through Docker ( Merkel, 2014) and Singularity ( Kurtzer *et al.*, 2017).

## Metagenome assembly

The metagenome assembly was generated using MetaMDBG ( Benoit *et al.*, 2024). The resulting bin sets of each binning algorithm were optimised and refined using MAGScoT ( Rühlemann *et al.*, 2022). PROKKA ( Seemann, 2014) was used to identify tRNAs and rRNAs in each bin, CheckM ( Parks *et al.*, 2015) (checkM_DB release 2015-01-16) was used to assess bin completeness/contamination, and GTDB-Tk ( Chaumeil *et al.*, 2022) (GTDB release 214) was used to taxonomically classify bins. Taxonomic replicate bins were identified using dRep ( Olm *et al.*, 2017) with default settings (95% ANI threshold). All bins were assessed for quality and categorised as metagenome-assembled genomes (MAGs) if they met the following criteria: contamination ≤5%, presence of 5S, 16S, and 23S rRNA genes, at least 18 unique tRNAs, and either ≥90% completeness or ≥ 50% completeness with fully circularised chromosomes ( Bowers *et al.*, 2017). Bins that did not meet these thresholds, or were identified as taxonomic replicates of MAGs, were retained as ‘binned metagenomes’ provided they had ≥50% completeness and ≤ 10% contamination.

## Genome sequence report

### Sequence data

**Figure 2.**
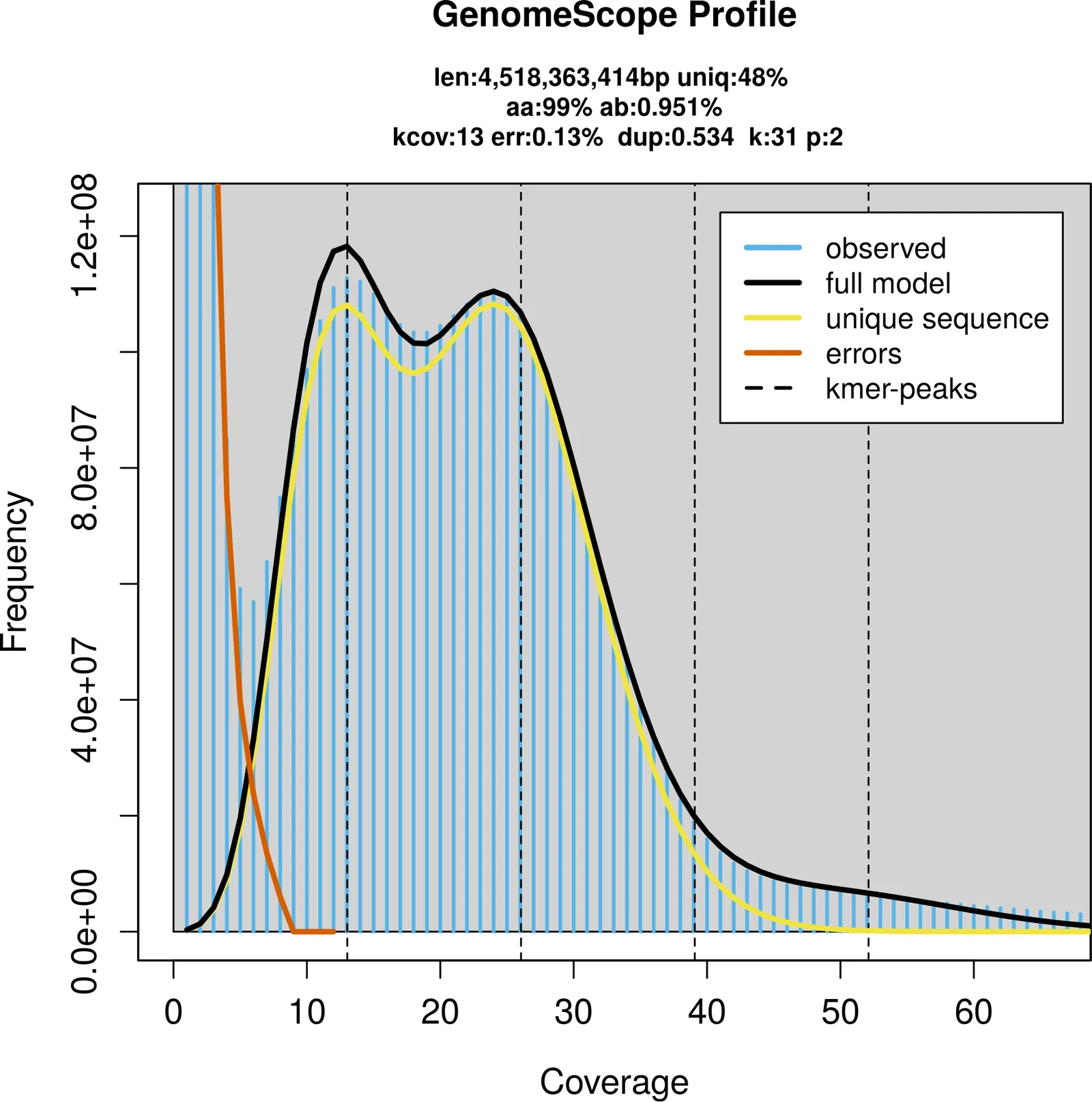
Frequency distribution of *k*-mers generated using GenomeScope2. The plot shows observed and modelled *k*-mer spectra, providing estimates of genome size, heterozygosity, and repeat content based on unassembled sequencing reads.

PacBio sequencing of the *Nausithoe racemosa* specimen generated 125.95 Gb (gigabases) from 16.42 million reads, which were used to assemble the genome. GenomeScope2.0 analysis estimated the haploid genome size at 4 540.17 Mb, with a heterozygosity of 0.95% and repeat content of 52.03% ( Figure 2). These estimates guided expectations for the assembly. Based on the estimated genome size, the sequencing data provided approximately 26× coverage. Hi-C sequencing produced 125.32 Gb from 414.96 million reads, which were used to scaffold the assembly. RNA sequencing data were also generated and are available in public sequence repositories. Table 1 summarises the specimen and sequencing details.

**Table 1.** Specimen and sequencing data for *Nausithoe racemosa* (BioProject PRJEB83148).

Platform	PacBio HiFi	Hi-C	RNA-seq
**ToLID**	jsNauRace1	jsNauRace1	jsNauRace1
**Specimen ID**	UCALI0000003	UCALI0000003	UCALI0000003
**BioSample (source individual)**	SAMEA112358969	SAMEA112358969	SAMEA112358969
**BioSample (tissue)**	SAMEA112359016	SAMEA112359018	SAMEA112359012
**Instrument**	Revio	Illumina NovaSeq X	Illumina NovaSeq X
**Run accessions**	ERR14048091; ERR14048092	ERR14056192	ERR14986724
**Read count total**	16.42 million reads	414.96 million read pairs	37.58 million read pairs
**Base count total**	125.95 Gb	125.32 Gb	11.35 Gb

### Assembly statistics

The genome was assembled into two haplotypes using Hi-C phasing. Haplotype 1 was curated to chromosome level, while haplotype 2 was assembled to scaffold level. The final assembly has a total length of 4 784.66 Mb in 3 010 scaffolds, with 5 452 gaps, and a scaffold N50 of 224.85 Mb ( Table 2).

**Table 2.** Genome assembly statistics for *Nausithoe racemosa.*

Genome assembly	Haplotype 1	Haplotype 2
**Assembly name**	jsNauRace1.hap1.1	jsNauRace1.hap2.1
**Assembly accession**	GCA_965183845.1	GCA_965183795.1
**Assembly level**	chromosome	scaffold
**Span (Mb)**	4 784.66	4 868.20
**Number of chromosomes**	20	-
**Number of contigs**	8 462	9 322
**Contig N50**	1.78 Mb	1.61 Mb
**Number of scaffolds**	3 010	3 564
**Scaffold N50**	224.85 Mb	220.4 Mb
**Longest scaffold length (Mb)**	364.8	366.94
**Organelles**	Mitochondrial genome: 13.97 kb	-

The scaffold count follows the NCBI assembly statistics and excludes organelle assemblies, which are reported separately where present.

**Figure 3.**
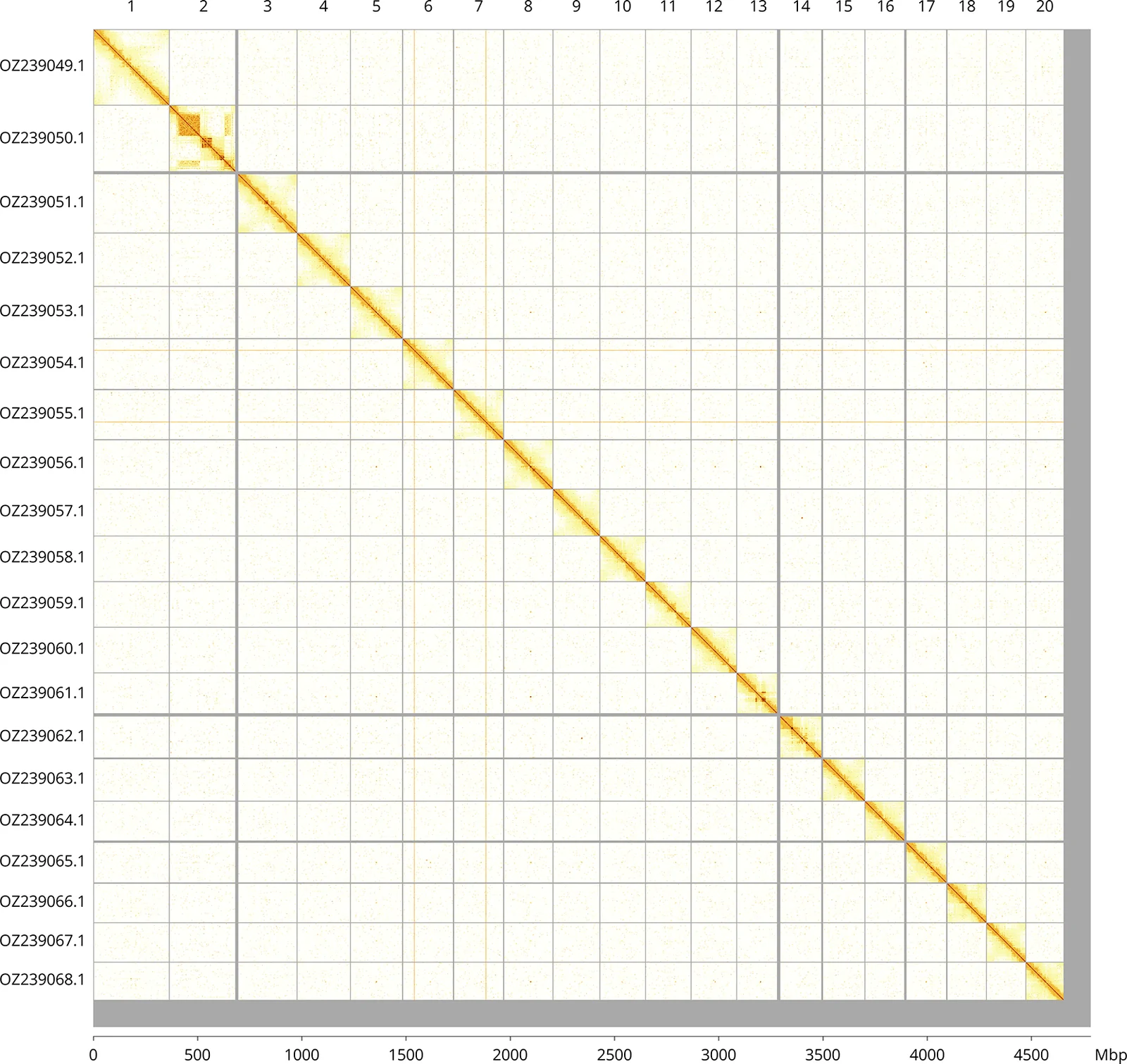
Hi-C contact map of the *Nausithoe racemosa* genome assembly. Assembled chromosomes are shown in order of size and labelled along the axes, with a megabase scale shown below. The plot was generated using PretextSnapshot.

Most of the haplotype 1 assembly sequence (97.34%) was assigned to 20 chromosomal-level scaffolds. These chromosome-level scaffolds, confirmed by Hi-C data, are named according to size ( Figure 3; Table 3). During genome curation, we noted that there are haplotypic inversions on Chromosome 1 (~343.43–356.71 Mb) and Chromosome 9 (~129.07–123.38 Mb). The order and orientation of scaffolds are uncertain in the following regions: Chromosome 2 (~159.59–197.12 Mb) and Chromosome 13 (~128.05–135.50 Mb).

**Table 3.** Chromosomal pseudomolecules in the haplotype 1 genome assembly of *Nausithoe racemosa* jsNauRace1.

INSDC accession	Molecule	Length (Mb)	GC%
OZ239049.1	1	364.80	37
OZ239050.1	2	329.92	36.50
OZ239051.1	3	282.21	37
OZ239052.1	4	256.11	37
OZ239053.1	5	251.82	37
OZ239054.1	6	244.29	37
OZ239055.1	7	240.12	37
OZ239056.1	8	235.79	37
OZ239057.1	9	224.85	37
OZ239058.1	10	219.25	37
OZ239059.1	11	218.79	37
OZ239060.1	12	218.73	37
OZ239061.1	13	206.82	36.50
OZ239062.1	14	205.47	37
OZ239063.1	15	202.20	37
OZ239064.1	16	197.93	37
OZ239065.1	17	196.24	37
OZ239066.1	18	189.37	37
OZ239067.1	19	188.17	37
OZ239068.1	20	184.39	37

The mitochondrial genome was also assembled (length 13.97 kb, OZ239069.1). This sequence is included as a contig in the multifasta file of the genome submission and as a standalone record.

### Assembly quality metrics

**Figure 4.**
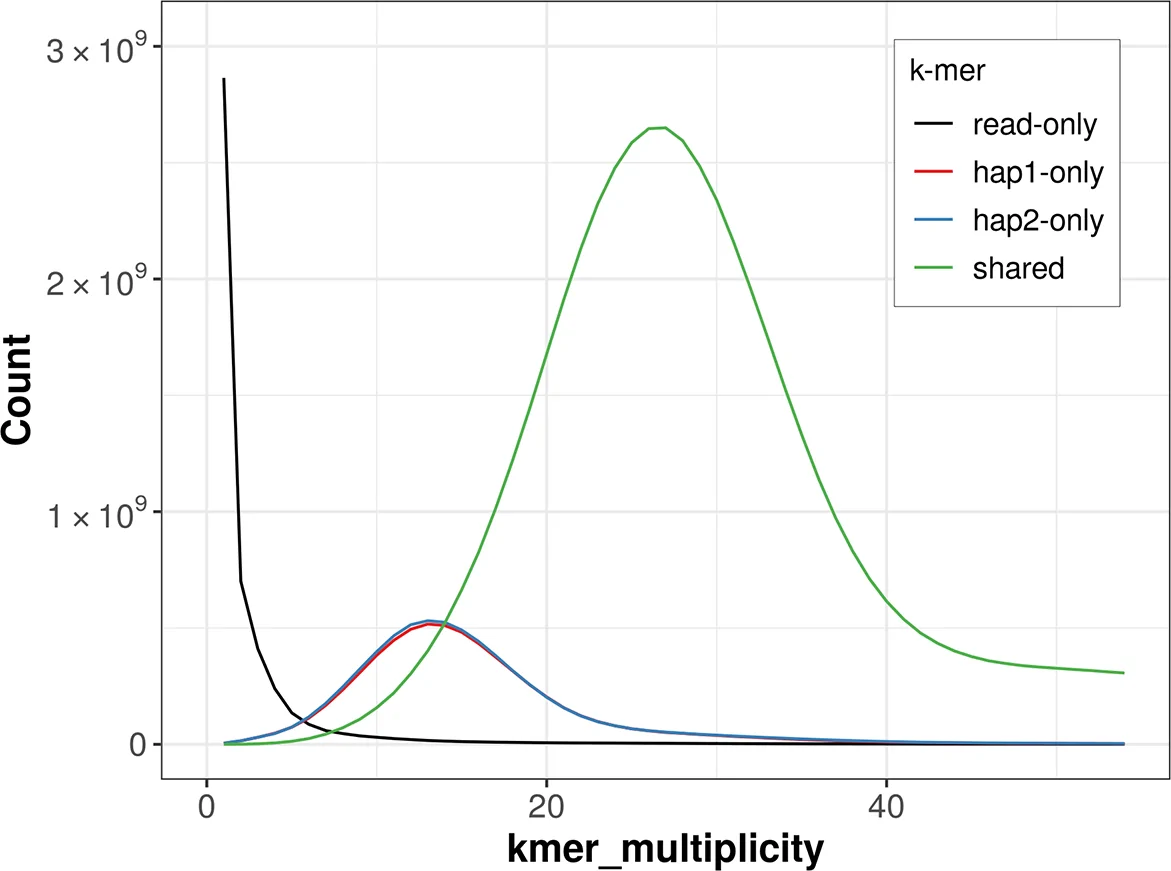
Evaluation of *k*-mer completeness using MerquryFK. This plot illustrates the recovery of *k*-mers from the original read data in the final assemblies. The horizontal axis represents *k*-mer multiplicity, and the vertical axis shows the number of *k*-mers. The black curve represents *k*-mers that appear in the reads but are not assembled. The green curve corresponds to *k*-mers shared by both haplotypes, and the red and blue curves show *k*-mers found only in one of the haplotypes.

For haplotype 1, the estimated QV is 60.1, and for haplotype 2, 59.8. When the two haplotypes are combined, the assembly achieves an estimated QV of 59.9. The *k*-mer completeness is 84.19% for haplotype 1, 84.65% for haplotype 2, and 98.91% for the combined haplotypes ( Figure 4).

**Figure 5.**
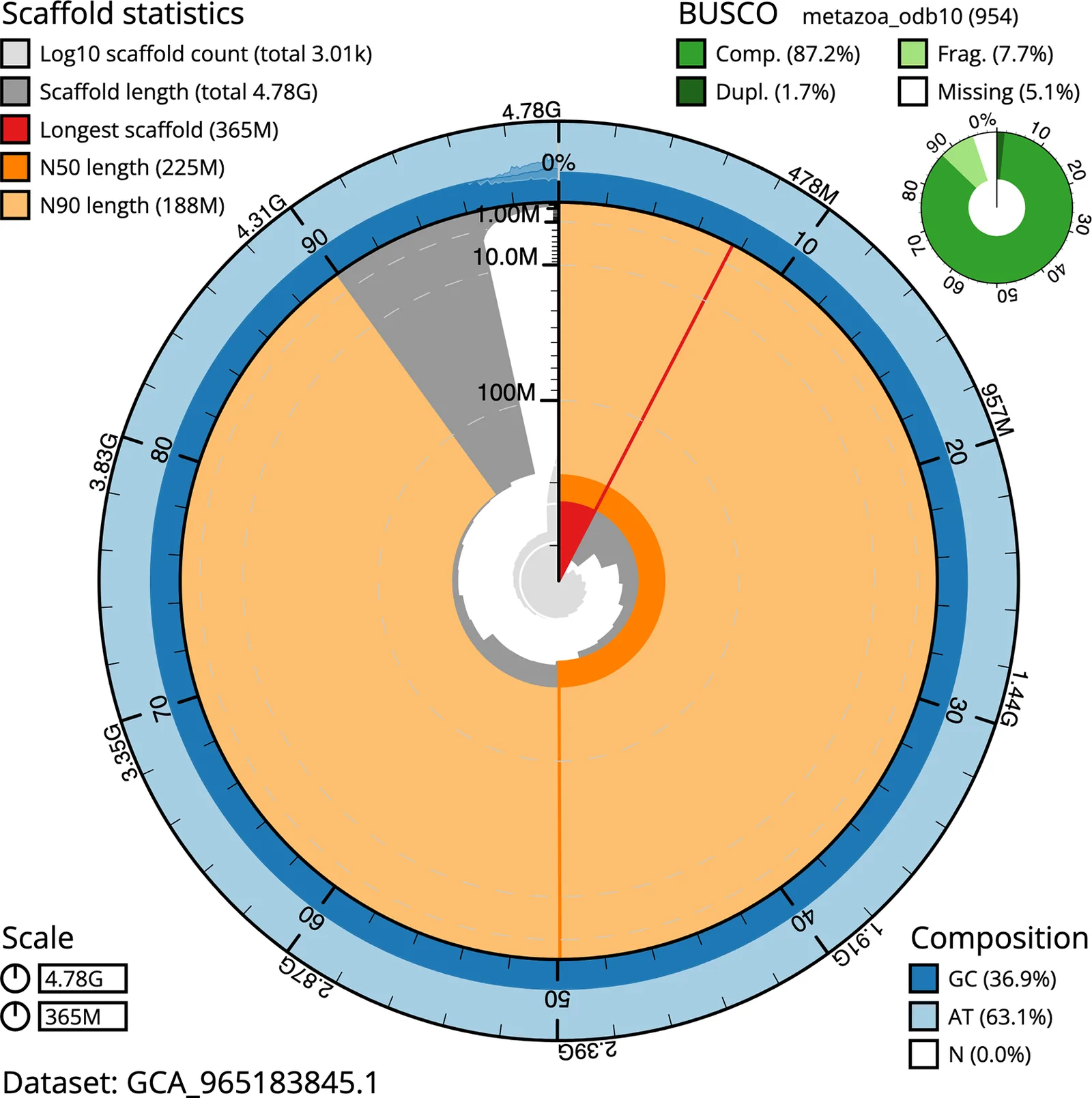
Assembly metrics for jsNauRace1.hap1.1. The BlobToolKit snail plot provides an overview of assembly metrics and BUSCO gene completeness. The circumference represents the length of the whole genome sequence, and the main plot is divided into 1 000 bins around the circumference. The outermost blue tracks display the distribution of GC, AT, and N percentages across the bins. Scaffolds are arranged clockwise from longest to shortest and are depicted in dark grey. The longest scaffold is indicated by the red arc, and the deeper orange and pale orange arcs represent the N50 and N90 lengths. A light grey spiral at the centre shows the cumulative scaffold count on a logarithmic scale. A summary of complete, fragmented, duplicated, and missing BUSCO genes in the set is presented at the top right. An interactive version of this figure can be accessed on the BlobToolKit viewer.

**Figure 6.**
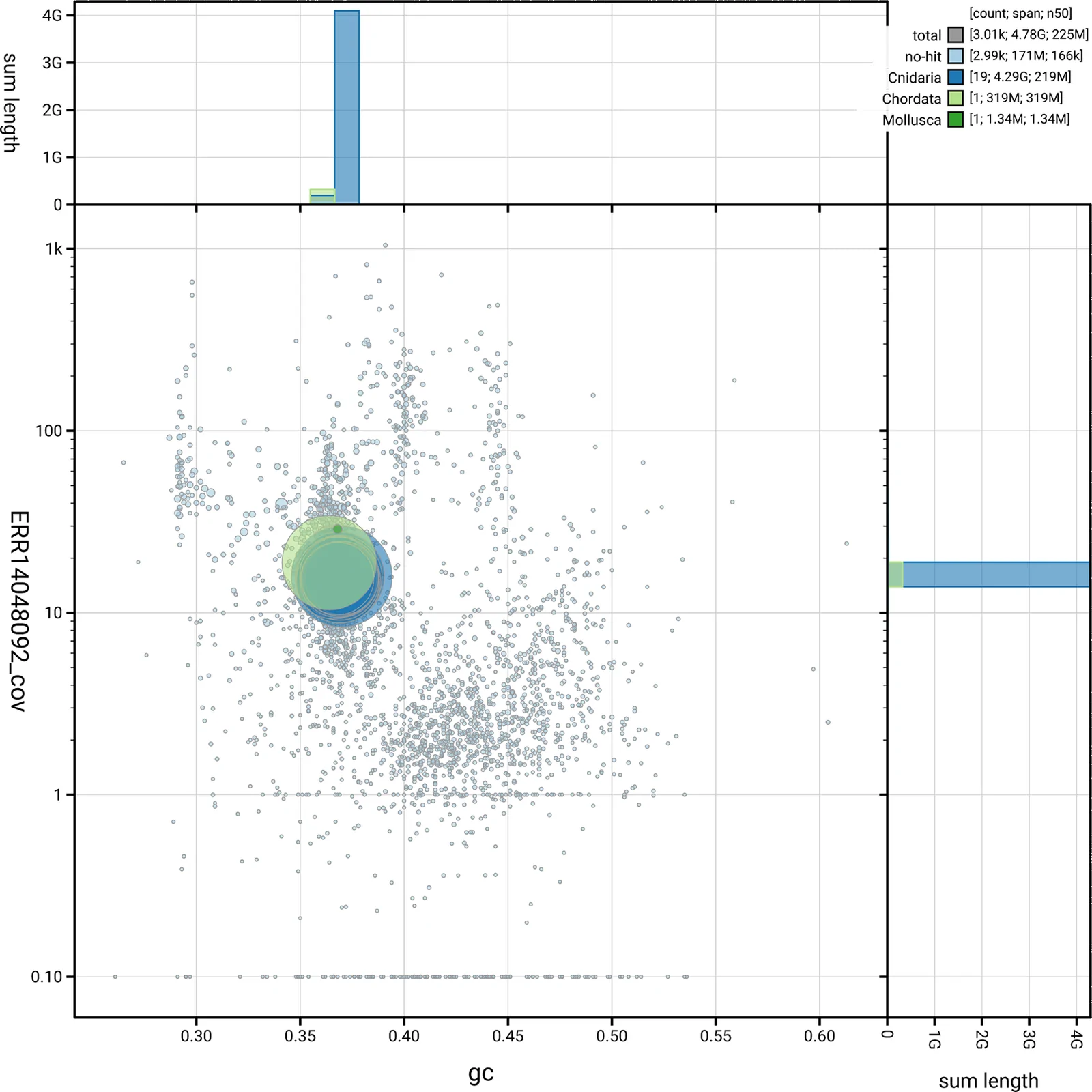
BlobToolKit blob plot for jsNauRace1.hap1.1. The plot shows base coverage (vertical axis) and GC content (horizontal axis). The circles represent scaffolds, with the size proportional to scaffold length and the colour representing phylum membership. The histograms along the axes display the total length of sequences distributed across different levels of coverage and GC content. An interactive version of this figure is available on the BlobToolKit viewer.

BUSCO analysis using the metazoa_odb10 reference set ( *n* = 954) identified 87.2% of the expected gene set (single = 85.5%, duplicated = 1.7%) in haplotype 1. For haplotype 2, BUSCO v.5.7.1 analysis identified 87.7% of the expected gene set (single = 85.7%, duplicated = 2.0%). The snail plot in Figure 5 summarises the scaffold length distribution and other assembly statistics for haplotype 1. The blob plot in Figure 6 shows the distribution of scaffolds by GC proportion and coverage for haplotype 1.

Table 4 lists the assembly metric benchmarks adapted from Rhie *et al.* (2021) and the Earth BioGenome Project Report on Assembly Standards January 2026. The EBP metric, calculated for haplotype 1, is **6.C.Q60**, meeting the recommended 6.C.Q40 reference standard.

**Table 4.** Earth Biogenome Project summary metrics for the *Nausithoe racemosa* assembly.

Measure	Value	Benchmark
EBP summary (haplotype 1)	6.C.Q60	6.C.Q40
Contig N50 length	1.78 Mb	≥ 1 Mb
Scaffold N50 length	224.85 Mb	= chromosome N50
Consensus quality (QV)	Haplotype 1: 60.1; haplotype 2: 59.8; combined: 59.9	≥ 40
*k*-mer completeness	Haplotype 1: 84.19%; Haplotype 2: 84.65%; combined: 98.91%	≥ 90%
BUSCO	Haplotype 1: C:87.2% [S:85.5%, D:1.7%], F:7.7%, M:5.1%, n:954; Haplotype 2: C:87.7% [S:85.7%, D:2.0%], F:7.0%, M:5.2%, n:954	S > 90%; D < 5%
Percentage of assembly assigned to chromosomes	97.34%	≥ 90%

**Notes:** The EBP summary uses log10(Contig N50); chromosome-level (C) or log10(Scaffold N50); Q (Merqury QV). BUSCO: C = complete; S = single-copy; D = duplicated; F = fragmented; M = missing; n = orthologues.

**Table 5.** Software versions and sources used for *Nausithoe racemosa.*

Software	Version	Source
BLAST	2.14.0	ftp://ftp.ncbi.nlm.nih.gov/blast/executables/blast+/
BlobToolKit	4.4.5	https://github.com/blobtoolkit/blobtoolkit
BUSCO	5.7.1	https://gitlab.com/ezlab/busco
bwa-mem2	2.2.1	https://github.com/bwa-mem2/bwa-mem2
CheckM	2015-01-16	https://ecogenomics.github.io/CheckM/
DIAMOND	2.1.8	https://github.com/bbuchfink/diamond
dRep	3.4.0	https://github.com/MrOlm/drep
fasta_windows	0.2.4	https://github.com/tolkit/fasta_windows
FastK	1.1	https://github.com/thegenemyers/FASTK
GenomeScope2.0	2.0.1	https://github.com/tbenavi1/genomescope2.0
Gfastats	1.3.6	https://github.com/vgl-hub/gfastats
GTDB-Tk	1.2.1	https://github.com/Ecogenomics/GTDBTk
Hifiasm	0.19.8-r603	https://github.com/chhylp123/hifiasm
HiGlass	1.13.4	https://github.com/higlass/higlass
MAGScoT	1.0.0	https://github.com/ikmb/MAGScoT
MerquryFK	1.1.0-c1	https://github.com/thegenemyers/MERQURY.FK
MetaMDBG	Pre-release	https://github.com/GaetanBenoitDev/metaMDBG
Minimap2	2.24-r1122; 2.28-r1209	https://github.com/lh3/minimap2
MitoHiFi	3.2.2	https://github.com/marcelauliano/MitoHiFi
MultiQC	1.14; 1.17 and 1.18	https://github.com/MultiQC/MultiQC
Nextflow	23.10.0; 24.10.4	https://github.com/nextflow-io/nextflow
PretextSnapshot	0.0.4	https://github.com/sanger-tol/PretextSnapshot
PretextView	1.0.3	https://github.com/sanger-tol/PretextView
Prokka	1.14.5	https://github.com/tseemann/prokka
samtools	1.17; 1.18; 1.2; 1.21; 1.6	https://github.com/samtools/samtools
sanger-tol/ascc	0.1.0	https://github.com/sanger-tol/ascc
sanger-tol/blobtoolkit	v0.7.1	https://github.com/sanger-tol/blobtoolkit
sanger-tol/curationpretext	1.4.2	https://github.com/sanger-tol/curationpretext
Seqtk	1.4-r122	https://github.com/lh3/seqtk
Singularity	3.9.0	https://github.com/sylabs/singularity
TreeVal	1.1.1	https://github.com/sanger-tol/treeval
YaHS	1.2a.2	https://github.com/c-zhou/yahs

### Metagenome report

We recovered one MAG from the metagenome assembly (GCA_965240435.1). It was assigned as *Moorena producens* (NCBI taxid:1155739), with a total genome size of 9.78 Mbp. The completeness was estimated to be 98.7% and the contamination 1.3%.

## Author information

Contributors are listed at the following links:

• Members of the Wellcome Sanger Institute Tree of Life Management, Samples and Laboratory team
• Members of Wellcome Sanger Institute Scientific Operations – Sequencing Operations
• Members of the Wellcome Sanger Institute Tree of Life Core Informatics team
• Members of the EBI Aquatic Symbiosis Genomics Data Portal Team
• The Aquatic Symbiosis Genomics Project leadership

## Wellcome Sanger Institute – legal and governance

The materials that have contributed to this genome note have been supplied by a Tree of Life collaborator. The Wellcome Sanger Institute employs a process whereby due diligence is carried out proportionate to the nature of the materials themselves, and the circumstances under which they have been/are to be collected and provided for use. The purpose of this is to address and mitigate any potential legal and/or ethical implications of receipt and use of the materials as part of the research project, and to ensure that in doing so we align with best practice wherever possible.

The overarching areas of consideration are:

• Ethical review of provenance and sourcing of the material
• Legality of collection, transfer and use (national and international)

Each transfer of samples is undertaken according to a Research Collaboration Agreement or Material Transfer Agreement entered into by the Tree of Life collaborator, Genome Research Limited (operating as the Wellcome Sanger Institute) and in some circumstances other Tree of Life collaborators.

## Data Availability

European Nucleotide Archive: Nausithoe racemosa. Accession number PRJEB83148; https://identifiers.org/ena.embl/PRJEB83148. The genome sequence is released openly for reuse. The *Nausithoe racemosa* genome sequencing initiative is part of the Aquatic Symbiosis Genomics Project (PRJEB43743) and the Sanger Institute Tree of Life Programme (PRJEB43745). All raw sequence data and the assembly have been deposited in INSDC databases. The genome will be annotated using available RNA-Seq data and presented through the Ensembl pipeline at the European Bioinformatics Institute. Raw data and assembly accession identifiers are reported in Tables 1 and 2. Production code used in genome assembly at the WSI Tree of Life is available at https://github.com/sanger-tol . Table 5 lists software versions used in this study.
