# Supplementary material for: The genome sequence of a coronate scyphozoan jellyfish, Nausithoe racemosa (Komai, 1936) (Coronatae: Nausithoidae), and a metagenome-assembled genome of the associated cyanobacterium Moorena producens
Source: Wellcome Open Res. 2026 Jul 28;11:476. [Version 1] doi: 10.12688/wellcomeopenres.27239.1 (PMC13562936; doi:10.12688/wellcomeopenres.27239.1)
Supplement: Peer review history

# Reviewer report: Reviewer response for version 1

**DOI:** 10.21956/wellcomeopenres.29980.r163078

**Published:** 2026-09-03

**Authors:** Shawn M Burgess

**Affiliations:** 1 National Human Genome Research Institute, Bethesda, USA

The manuscript: *The genome sequence of a coronate scyphozoan jellyfish, Nausithoe racemosa (Komai, 1936) (Coronatae: Nausithoidae), and a metagenome-assembled genome of the associated cyanobacterium Moorena producens *describes the effort to sequence and assemble the genome of a jellyfish that resides in a somewhat uncertain location in the evolutionary tree. The paper is clear and concise on how the genome was generated and uses state of the art approaches to generate a high-quality annotated genome.

 The only comment I have regarding the manuscript is that the introduction is a bit hard to follow in terms of how the organism sits in the clade in relation to other closely related jellyfish. I think it would be helpful (but not an absolute requirement for “acceptance”) if included in figure 1 there was a tree showing the possible alternative relationships of these jellyfish or the branches in question. Otherwise, I have no other objections.

Are sufficient details of methods and materials provided to allow replication by others?

Yes

Is the rationale for creating the dataset(s) clearly described?

Yes

Are the datasets clearly presented in a useable and accessible format?

Yes

Are the protocols appropriate and is the work technically sound?

Yes

Reviewer Expertise:

NA

I confirm that I have read this submission and believe that I have an appropriate level of expertise to confirm that it is of an acceptable scientific standard.

# Reviewer report: Reviewer response for version 1

**DOI:** 10.21956/wellcomeopenres.29980.r163080

**Published:** 2026-08-31

**Authors:** Nicholas S Kron

**Affiliations:** 1 Department of Marine Biology and Ecology, Rosenstiel School of Marine, Atmospheric, and Earth Science, University of Miami, Miami, Florida, USA

**Reviewer summary:**

 This report by Dawson et al. describes the sampling, sequencing, and assembly of a chromosome level genome for the scyphozoan *Nausithoe racemosa* which lacks genomic resources. The authors describe the paucity of sufficient resources in this clade of (Coronatae) to answer questions regarding phylogeny and the evolution of photosymbiosis in the clade. The authors describe the collection of samples from wild caught jellies in Palau and their subsequent sampling and sequencing at the Wellcome Sanger Institute using standard protocols.  The genome was assembled from PacBio HiFi reads and Hi-C data with Hifiasm in phasing mode and then scaffolded with Hi-C reads followed by automatd and manual curation with ToL standard pipelines, tools, and workflows. The resulting assembly was at the chromosome scale (20 pseudo-molecules) with a BUSCO completeness at ~87% for metazoa odb10. The author also describe collection of RNAseq reads, assembly of the mitochondrion, and metagenome assembly of 1 symbiont, but do not present analysis or results for these beyond estimated completeness for the one identified MAG that passed quality metrics.  

**Is the rationale for creating the dataset(s) clearly described?**

 Yes. The authors outline a clear paucity of available genetic resources and several specific questions creating this resource could answer.

**Are the protocols appropriate and is the work technically sound?**

 Overall, yes. Protocols described are clear and comprise standard tools and workflows. The estimated 26X coverage is a bit on the low end for high-quality reference genome, but this is to be expected when working with hard to collect non-models.

 My one major concern is regarding endosymbionts. The introduction notes that this species is known to carry Symbiodiniaceae as endosymbionts. In other photosymbiotic cnidarians, these can represent a significant fraction of total reads (up to ~1/3 in corals). Is this also the case with this species? Was the life stage collected not generally associated with this symbiont? The authors do not describe decontamination of dinoflagellate reads prior to assembly, so I assume these were included in the initial 26X estimate from GenomeScope and assembled in the primary assembly and purged downstream with ASCC. The authors describe how much manual curation affected assemblies, but do not describe the results of the ASCC run. What fraction of raw reads and/or contigs were purged as contaminants? The final BlobTools plot does suggests successful purging of any symbiont material. This is also relevant to the metagenome assembly, as the MAG methods appear to target only prokaryote lineages. Given that the primary known endosymbiont is a Eukaryote, the major endosymbiont would be missed. Given that the introduction centers the photosymbiotic nature of this jelly and its specific symbiont, I would like to see this aspect of the assembly addressed (how much symbiont material was recovered, what was done with it, how that impacted the assembly, etc.).

**Are sufficient details of methods and materials provided to allow replication by others?**

 For the majority, yes. The tools, versions, and order in which were used are thoroughly documented which is appreciated.

 The authors mention in paragraph 1 of ‘Genome assembly’ that they used ‘filtered reads’ as input into GenomeScope2 but no prior section describes any form of raw read filtering or QC. Are we to assume this was handled in the SMRT Link primary and secondary data analysis mentioned in the ‘PacBio HiFi library preparation and sequencing’ section?

 Some minor additional notes for this question:

- The authors assess genome completion with metazoa_odb10. This is acceptable for comparison to metazoa genomes at large, but there are more recent and lineage specific BUSCO databases available. While not always strictly followed in the literature, BUSCO best practice is to use the most recent and specific lineage possible, which here would be cnidaria_odb12. Inclusion of BUSCO assessment to this database would provide a better sense of completeness.
- The authors do not specify whether default parameters were used. Although this is generally assumed when no specification is provided, it would be good to specify that default params were used and why.
- The authors also do not specify the versions/tags of Docker containers used which would also be appreciated for reproducibility although not strictly necessary.  

**Are the datasets clearly presented in a useable and accessible format?**

 Yes. The author list exact accessions for the raw data and assemblies in public repositories.

**Additional notes**

- The authors describe the collection of RNA and sequencing of said RNA. While this report is a genome assembly report, not a full resource report with transcriptome and annotation, it would be nice to see the fraction of read alignment to the final assembly as another metric of completeness.
- The authors note a mitochondrial assembly with MitoHiFi. They report it is included in the final assembly and as a stand-alone record. I am not familiar with the Wellcome Sanger Institute standard for reporting genomes (e.g. if the mitochondrial assembly is customarily not discussed since it is not part of the nuclear genome) but given the mitochondrial assembly is reported it would be nice to at least see the MitoHiFi report and annotation results (especially since there is a metagenome report as well).

**Overall assessment**

 The authors present a very thorough report and generally high-quality assembly for this under sampled clade of marine organisms. This resource will undoubtedly be very useful. Barring some questions regarding the Symbiodiniaceae endosymbiont, this report is ready for indexing. Well done authors.

Are sufficient details of methods and materials provided to allow replication by others?

Partly

Is the rationale for creating the dataset(s) clearly described?

Yes

Are the datasets clearly presented in a useable and accessible format?

Yes

Are the protocols appropriate and is the work technically sound?

Partly

Reviewer Expertise:

genome assembly and annotation, transcriptomics

I confirm that I have read this submission and believe that I have an appropriate level of expertise to confirm that it is of an acceptable scientific standard, however I have significant reservations, as outlined above.

# Reviewer report: Reviewer response for version 1

**DOI:** 10.21956/wellcomeopenres.29980.r163075

**Published:** 2026-08-06

**Authors:** Bingpeng Xing

**Affiliations:** 1 Third Institute of Oceanography Ministry of Natural Resources, Xiamen, Fujian, China

The authors report a haplotype-resolved, chromosome-level genome assembly of the coronate scyphozoan *Nausithoe racemosa*. The two haplotypes are approximately 4.78 and 4.87 Gb in size, respectively, with 97.34% of haplotype 1 anchored to 20 chromosome-level pseudomolecules. The assembly QV is close to 60, and the combined k-mer completeness of the two haplotypes is 98.91%. These data represent a valuable genomic resource for research on Coronatae, the family Nausithoidae, and comparative genomics of cnidarians.

 The principal concerns with the current manuscript are as follows:

 The evidence supporting host species identification and the information on voucher specimens are insufficient. It is unclear whether the identification was based on the medusa stage, the polyp stage, or life-history stages obtained through laboratory culture. The manuscript also does not state whether molecular identification using markers such as COI, 16S, 18S, or 28S was performed.

 The description of the cyanobacterium as “associated” in the title is not supported by direct evidence.

 Table 1 lists only the host PacBio HiFi, Hi-C, and RNA-seq datasets, without identifying a separate dataset corresponding to the reported “metagenome data.”

 There is an apparent inconsistency between the reported GTDB-Tk software version and the GTDB database release.

 The description and quality assessment of the MAG are far less comprehensive than would be expected for a dataset that constitutes a major component of the article title.

 Finally, the presentation of the host genome quality is somewhat selective. The manuscript does not adequately explain why the BUSCO completeness and haplotype-specific k-mer completeness values fail to meet the benchmarks listed in the corresponding table.

Are sufficient details of methods and materials provided to allow replication by others?

Partly

Is the rationale for creating the dataset(s) clearly described?

Partly

Are the datasets clearly presented in a useable and accessible format?

Partly

Are the protocols appropriate and is the work technically sound?

Partly

Reviewer Expertise:

genome，DNA barcoding，mitogenome，marine biodiversity

I confirm that I have read this submission and believe that I have an appropriate level of expertise to confirm that it is of an acceptable scientific standard, however I have significant reservations, as outlined above.
